# Electronic Structure of Pentagonal Carbon Nanocones: An ab Initio Study

**DOI:** 10.1021/acs.jpca.3c05062

**Published:** 2023-11-08

**Authors:** Samuel Henrique Mattoso, Véronique Brumas, Stefano Evangelisti, Giovanna Fronzoni, Thierry Leininger, Mauro Stener

**Affiliations:** † Dipartimento di Scienze Chimiche e Farmaceutiche, University of Trieste, Via Giorgieri 1, 34127 Trieste, Italy; ‡ Laboratoire de Chimie et Physique Quantiques - FeRMI, Université de Toulouse 3 (Paul Sabatier) et CNRS, 118, Route de Narbonne, F-31062 Toulouse, Cedex, France

## Abstract

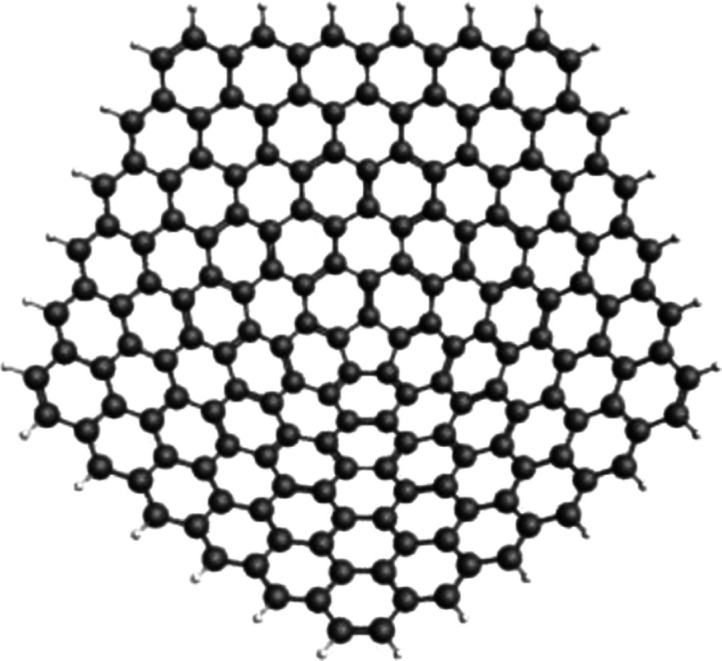

In this work, we investigate the electronic structure of a particular class of carbon nanocones having a pentagonal tip and *C*_5*v*_ symmetry. The ground-state nature of the wave function for these structures can be predicted by the recently proposed generalized Hückel rule that extends the original Hückel rule for annulenes to this class of carbon nanocones. In particular, the structures here considered can be classified as closed-shell or anionic/cationic closed-shells, depending on the geometric characteristics of the cone. The goal of this work is to assess the relationship between the electronic configuration of these carbon nanocones and their ability to gain or lose an electron as well as their adsorption capability. For this, the geometry of these structures in the neutral or ionic forms, as well as systems containing either one lithium or fluorine atom, was optimized at the DFT/B3LYP level. It was found that the electron affinity, ionization potential, and the Li or F adsorption energy present an intimate connection to the ground-state wave function character predicted by the generalized Hückel rule. In fact, a peculiar oscillatory energy behavior was discovered, in which the electron affinity, ionization energy, and adsorption energies oscillate with an increase in the nanocone size. The reasoning behind this is that if the anion is closed-shell, then the neutral nanocone will turn out to be a good electron acceptor, increasing the electron affinity and lithium adsorption energy. On the other hand, in the case of a closed-shell cation, this means that the neutral nanocone will easily lose an electron, leading to a smaller ionization potential and higher fluorine adsorption energy.

## Introduction

Carbon is a very unique element due to its ability to form long covalently bonded chains made up of only carbon atoms. Until recently, diamond and graphite were thought to be the only two stable structures found naturally, and they present widely different properties. Diamond is a hard, transparent insulator, while graphite is a black, soft, and poorly conducting lubricant material.^1^ This fact shows that two carbon materials can have fundamentally different properties due to a difference in structure and bonding. Surprisingly, these two structures are not the only possible carbon allotropes, and a whole series of other possible edifices exists. The scientific understanding of carbon structures fundamentally changed when fullerenes, also known as carbon buckyballs, were discovered in 1985.^2^ Other unique carbon nanostructures were discovered after that, such as carbon nanotubes (CNTs) in 1991 and graphene in 2004.^3,4^ These diverse synthetic carbon allotropes are part of a growing family of remarkable structures with pleasing architectures and outstanding material properties.^5^ One form of carbon that has not received as much attention as its cousins is carbon nanocones (CNCs), which were first synthesized in 1994.^6^ These structures are formed by introducing geometrical defects into a graphene network, leading to the formation of nonplanar molecules. Due to the CNCs’ geometry, there are considerable spatial differences regarding their properties. The apical-cone chemistry closely resembles fullerenes, while the cone sidewall chemistry is more closely related to graphene and large-diameter nanotubes.^7,8^ In this context, CNCs have been studied for a wide range of applications, as alternatives to CNTs, such as energy storage, gas storage, sensing applications, and drug delivery.^9−13^ CNCs have two main advantages over CNTs: (i) they do not require a potentially toxic metal catalyst and (ii) can be mass produced at room temperatures.^7^

A particular type of CNCs was recently investigated in our group, both at the Hückel and ab initio levels.^14^ They are composed of graphene triangular fragments inserted on a central annulene. This is the case, for instance, for coronene and corannulene, composed of benzene rings surrounding a benzene and a cyclopentadiene molecule, respectively. Because corannulene can be seen as a prototype of these structures and because of the presence of graphene sectors around the central annulene, we named these structures, and, more generally, the whole family of related structures, with the term of graphannulenes (GA).^14^ In general, a graphannulene conical structure [GA_*n*_(0, *d*_o_)] is a molecular system composed of *n* graphene triangular sectors, each sector confining with the two neighboring ones on two sides. The result is a system having a regular conical shape, at least in the case *n* < 6. Notice, however, that if *n* = 6 we have a flat hexagonal fragment of graphene, while if *n* > 6, structures having more complex shapes are obtained. The resulting structure is therefore composed of a series of *d*_o_ + 1 concentric carbon rings (where *d*_o_ is a non-negative integer), labeled by an integer number *j* (with 0 ≤ *j* ≤ *d*_o_), each ring containing a total of *n*(*j* + 1) atoms. The more general structures are then obtained by “cutting the tip” of the cones. In this way, GA_*n*_(*d*_*i*_, *d*_o_) structures are obtained by deleting the *d*_*i*_ innermost carbon rings around the GA_*n*_(0, *d*_o_) apex and saturating the inner-border carbons by hydrogen atoms. Here, again, *d*_*i*_ and *d*_o_ are non-negative integers. Notice that the topological structure of a graphannulene is completely defined once the order *n* of the symmetry axis, and the two “topological distances” from the center, *d*_*i*_ and *d*_o_, are given. Because ring *j* contains *n*(*j* + 1) atoms, the total number *N* of carbons in GA_*n*_(*d*_*i*_, *d*_o_) is given by . The total number of hydrogen atoms, on the other hand, is given by *N*_H_ = *n*(*d*_*i*_ + (*d*_o_ + 1)), where the first term corresponds to the inner edge (if present), while the second term corresponds to the outer edge.

**Figure 1:**
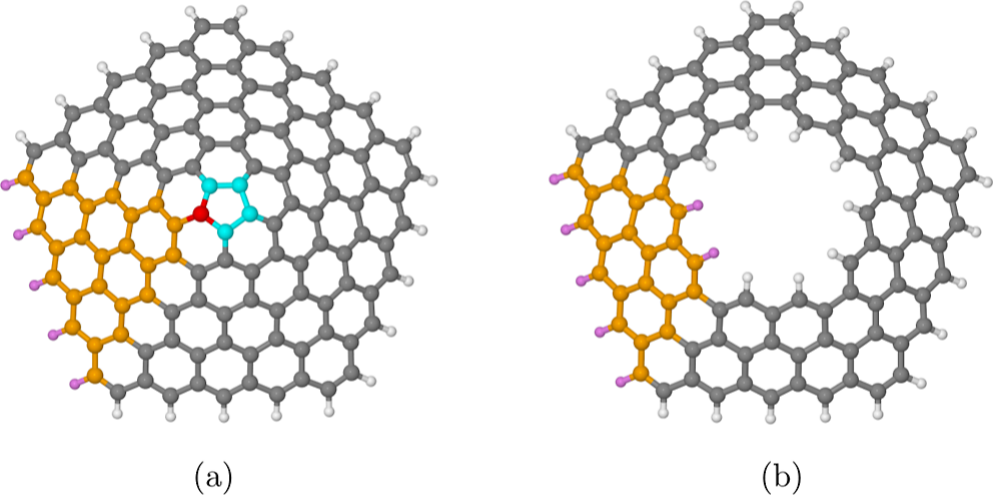
(a) Conical hat structure for *n* = 5 built by growing each triangular sector starting from the central pentagonal carbon ring. The latter is color-coded in cyan, and a single graphene triangle is highlighted in orange, with the corresponding connecting carbon atom of the central ring in red. Purple is used for the saturating hydrogen atoms in the triangular graphene sector. (b) Topological annulus, which, roughly speaking, corresponds to a truncated cone, for *n* = 5 built by deleting the two innermost rings of the complete cone structure reported in (a). Reproduced with permission from ref (14) Copyright 2021 American Chemical Society.

In this article, we focus our attention on graphannulenes of the type GA_5_(*d*_*i*_, *d*_o_), which are structures having a 5-fold symmetry axis. Two structures of this type are shown in Figure 1. In the case where *d*_*i*_ = 0, the cone has a single pentagon on the apex. This is illustrated on the left side of the figure, where GA_5_(0, 4) is shown. On the right side, we reported the structure of GA_5_(2, 4). As we discuss in detail in the next section, the GHR predicts for these structures a well-defined electronic structure at the Hückel level. In particular, they always have a closed-shell wave function when the number of carbon atoms is even (in other words, there are never partly occupied orbitals at the Fermi level). On the other hand, these structures obviously have a radical nature when *N*_C_ is odd. However, the radical structures are divided into two sets, according to the fact that they easily form cations or anions, respectively. These behaviors exhibited at the Hückel level are found also at the ab initio level. For instance, the ab initio ionization potential and electron affinity of the cones show an oscillatory behavior, in full agreement with the nature of the wave function predicted at the Hückel level.

This article is organized as follows. In Section 2, we briefly describe the generalized Hückel rule (GHR) for generic GA_*n*_(*d*_*i*_, *d*_o_) structures. In Section 3, we report the computational details of the numerical investigations. Section 4 reports the results and discussions concerning the numerical investigations. Finally, in Section 5, we draw some conclusions and discuss future works.

## Generalized Hückel Rule

We briefly discuss here, for the sake of completeness, the generalized Hückel rule for a generic GA_*n*_(*d*_*i*_, *d*_o_) graphannulene, and we focus our attention on the *n* = 5 structures, which are the object of the present investigation.

According to the original Hückel’s rule for aromaticity, closed single-ring conjugated π systems with a 4*N* + 2 number of π electrons, where N is an integer, are aromatic (i.e., with a closed-shell ground state). On the other hand, if these systems have a 4*N* number of π electrons, they are antiaromatic (systems with an open-shell ground state that corresponds to two electrons placed in two degenerate molecular orbitals). Here, aromaticity is understood as stability of the ring in comparison to its open-chain counterpart, whereas antiaromaticity is understood as instability, the source of this stability being due to the closed-shell wave function character. Although this fact is not often mentioned, it is important to notice that the aromaticity rule also applies to chains having an odd number of electrons: neutral radical molecules may become aromatic closed shells when losing or gaining an electron. In this case, we are in the presence of cationic closed-shell (CS^+^) or anionic closed-shell (CS^–^), corresponding to the 4*N* + 3 and 4*N* + 1 cases, respectively. A well-known example of these ionic systems is represented by the cyclopentadienyl anion (Cp^–^), the most common anion in organic chemistry. Finally, we would like to stress that, strictly speaking, the Hückel rule can be rigorously demonstrated only for model Hamiltonians, while in real systems many additional factors (geometric distortions, steric hindrance,···) can make the situation much more complex.

CNCs are complex structures that do not consist of just a single ring and the Hückel rule, in its original form, cannot be applied to them. Despite this simple fact, it is not rare to find in the scientific literature sentences that describe coronene or corannulene as being “exceptions” to the Hückel rule, which is simply incorrect. Additionally, it is important to note that when dealing with such complex structures, the notion of aromaticity becomes more complex and is not directly a synonym for stability anymore. Various structures that violate bonding and aromatic rules, such as certain triangulenes, have been synthesized; aromaticity, nevertheless, remains a useful quantity.^15^ For CNCs containing one annulene ring at the tip and closely related structures, a generalized Hückel rule has been proposed.^14^ These structures include as special cases coronene, corannulene, kekulene, and many others. They are characterized by a symmetry axis of order *n*, and the two topological indexes *d*_*i*_ and *d*_o_. The order *n* of the symmetry axis is an integer number going, in principle, from one to infinity, although, as already mentioned, real systems having *n* > 6 are geometrically distorted and an axis of symmetry is in general no longer present.

The GHR predicts the character of the ground state based on the three geometrical indexes that define this type of CNC. In order to establish the GHR, it is convenient to define the integer type of an integer number *d*: we say that , with *k* = 0, 1, 2, 3, if *d* can be expressed under the form *d* = 4*m* + *k*, where *m* is also an integer number. The GHR was described in detail previously;^14^ here, we summarize it in the general case of a GA_*n*_(*d*_*i*_, *d*_o_) graphannulene, the rule can be enunciated as follows:

1. The number of rings is even (and hence *d* = *d*_o_ – *d*_*i*_ is odd): the system is always a CS.
2. The number of rings is odd (and hence *d* = *d*_o_ – *d*_*i*_ is even): we have four cases:
  (a) OS
  (b) then:
    i. *d*_*i*_ and *d*_o_ are both even ⇒ CS^–^
    ii. *d*_*i*_ and *d*_o_ are both odd ⇒ CS^+^
  (c) CS
  (d) then:
    i. *d*_*i*_ and *d*_o_ are both even ⇒ CS^+^
    ii. *d*_*i*_ and *d*_o_ are both odd ⇒ CS^–^

In this work, we restrict our investigation on the case *n* = 5, which means that . Therefore, the GHR predicts the following three different cases:

1. If *d*_*i*_ and *d*_o_ have different parities, the wave function is a CS.
2. If *d*_*i*_ and *d*_o_ are both even, the wave function is of type CS^–^.
3. If *d*_*i*_ and *d*_o_ are both odd, the wave function is of type CS^+^.

Notice that in case 1 the GA structure contains an even number of concentric rings, while an odd number of rings is present in both cases 2 and 3. For this reason, GA of type 1 contains a total even number of carbon atoms, while in the case of both types 2 and 3 the number of carbon atoms is odd. Therefore, the neutral structures of types 2 and 3 are necessarily radicals.

Molecules to which the rule can be applied are, for instance, coronene [GA_6_(0, 1)], corannulene [GA_5_(0, 1)], and kekulene [GA_6_(1, 2)]. Because all these structures have an even number of rings, they are all closed-shells, regardless of the order of the symmetry axis *n* and the number of carbon atoms. At the moment, we have proved the GHR for any *n* only for the special case *d*_o_ = *d*_*i*_ + 1, although at the moment it seems very plausible that its validity is, for model Hamiltonians, completely general. Notice that the previously mentioned coronene, corannulene, and kekulene molecules belong precisely to the *d*_o_ = *d*_*i*_ + 1 case.

## Computational Methods

The CNCs were built by using the Avogadro software.^16^ A simple homemade FORTRAN90 program that computes the Cartesian coordinates of a given cone was also used. The code defines the coordinates of an ideal cone, and the structure so obtained is used as a guess geometry for subsequent optimization. The results of the optimized geometry, that is, the energies and bond lengths, were the same regardless of the initial guess geometry. All results that do not specify the method were obtained with a geometry optimization at the DFT level.

### Density Functional Theory

All DFT calculations were carried out using the Amsterdam density functional (ADF) program, which is part of the Amsterdam Modeling Suite (AMS).^17^ All results discussed take into consideration the optimized geometry of the neutral and ionic CNCs. Therefore, all of the electron affinities and ionization potentials are adiabatic. The B3LYP exchange and correlation energy functional was used for all calculations, in which ADF uses VWN5 in B3LYP (20% Hartree–Fock exchange).^18^ ADF uses Slater Type Orbitals (STOs) as basis functions and in this work, a double-ζ polarized (DZP) basis set was used for the carbon atoms, a double-ζ (DZ) was used for the hydrogens, and for Li and F a DZ basis set and an augmented AUG/ADZP basis set were used, respectively. The augmented AUG/ADZP basis consists of a DZ set plus polarization plus one diffuse s, p, and d functions.

In this work, the EAs and IPs were computed at the DFT level within the ΔSCF Kohn–Sham (ΔKS) scheme: the EA^ΔKS^ is given by the difference between the energy of the *N*-electron configuration and that of the *N* + 1 electronic configuration, while the IP^ΔKS^ is given by the difference between the energy of the *N* – 1 electronic configuration and that of the *N*-electron configuration. Both EA and IP were calculated at the adiabatic level, therefore considering the relaxation of the anionic and cationic geometrical structures of the nanocones. Only the lowest order calculated EAs and IPs have been considered.

The GHR was used to predict the ground-state wave function of the CNCs. In the case of the CS CNCs, the *C*_5*v*_ symmetry was used and restricted calculations were performed. In the case of CS^–^ or CS^+^ cones, which possess an open-shell wave function when neutral, unrestricted calculations were performed, in which the symmetry was not used and the coordinates were slightly modified in order to make the molecule asymmetric. This prevents the molecule’s geometry from being stuck at a local energy minimum and not correctly optimizing. Indeed, all open-shell CNC systems have a distorted geometry.

### Restricted Hartree–Fock and Coupled Cluster

The MOLPRO software package was used to carry out all coupled cluster (CC) and Hartree–Fock (HF) calculations.^19^ Initially, a restricted Hartree–Fock (RHF) calculation was performed, followed by a geometry optimization. After that, by using the RHF orbitals, a coupled cluster singles and doubles (CCSD) calculation was done, followed by a second geometry optimization performed at this level. Finally, a CCSD calculation with a perturbative treatment of triple excitations [CCSD(T)] was performed. The basis sets used for these calculations, besides the minimal STO-3G basis employed to get a qualitative picture of these systems, were atomic natural orbital (ANO) basis sets. In particular, the Roos “triple-ζ plus polarization” basis sets were used.^20^ These are atomic natural orbital (ANO) basis sets, whose primitive gaussians are (14s9p4d3f/8s4p3d) for (C/H), respectively. We used several spherical harmonics contractions of these basis sets: the 3s2p/2s (VDZ), 3s2p1d/2s1p (VDZP), and, finally, the complete (6s5p3d2f/8s4p3d) contractions suggested by the authors. We are well aware of the fact that a minimal (STO-3G) basis set is able to give only a qualitative agreement, and even a DZ basis set is not particularly accurate. However, we decided to report these results anyway because they show a general trend toward more accurate results.

## Results and Discussion

Two series of nanocones are considered in this study: the GA_5_(0, *q*) series with the pentagonal ring on the tip and the GA_5_(1, *q*) open cones series, obtained by removing the pentagon tip, in order to analyze how this removal affects the properties of the systems. According to the GHR, the wave function character of the GA_5_(0, *q*) cones is CS if q is odd and CS^–^ if q is even, while for the GA_5_(1, *q*) cone it is CS if q is even and CS^+^ if q is odd, as reported in Table 1. The number of carbon atoms in each cone structure is also shown, which corresponds to the number of π electrons of the system.

**Table 1:** CNC Systems in This Study, Chemical Formulas and Wave Function Character Predicted by the GHR

system	chemical formula	GHR
GA_5_(0, 1)	C_20_H_10_	CS
GA_5_(0, 3)	C_80_H_20_	CS
GA_5_(0, 4)	C_125_H_25_	CS^–^
GA_5_(0, 5)	C_180_H_30_	CS
GA_5_(0, 6)	C_245_H_35_	CS^–^
GA_5_(0, 7)	C_320_H_40_	CS
GA_5_(0, 8)	C_405_H_45_	CS^–^
GA_5_(0, 9)	C_500_H_50_	CS
GA_5_(1, 2)	C_40_H_20_	CS
GA_5_(1, 3)	C_75_H_25_	CS^+^
GA_5_(1, 4)	C_120_H_30_	CS
GA_5_(1, 5)	C_175_H_35_	CS^+^
GA_5_(1, 6)	C_240_H_40_	CS
GA_5_(1, 7)	C_315_H_45_	CS^+^

Several properties have been considered to characterize these systems and to establish possible connections between the property and the ground-state wave function characters, as predicted by the GHR. To this purpose, we investigate the main geometrical parameters of the CNCs, as well as electronic properties, such as HOMO–LUMO gap, cohesive energy, dipole moment, electron affinity, ionization potential, and adsorption energies.

### Geometrical and Electronic Structure

**Figure 2:**
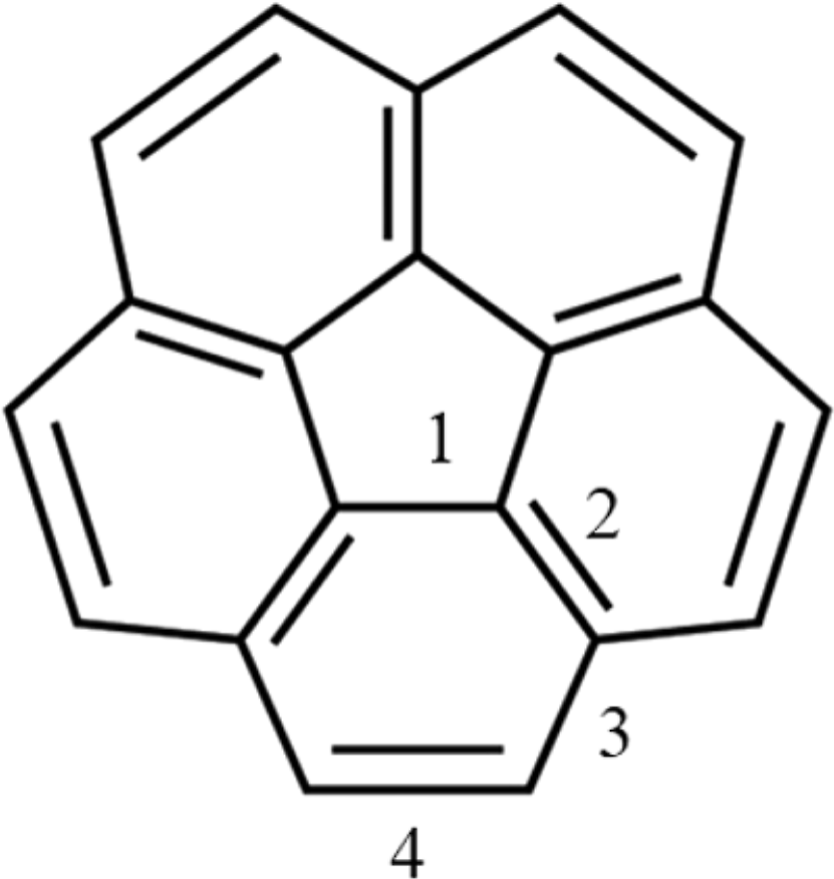
Corannulene shows its four unique C–C bonds. The double bonds show one of the many possible resonance structures.

The smallest curved case is given by GA_5_(0, 1), which corresponds to a bowl-shaped molecule known as corannulene, as reported in Figure 2.

GA_5_(0, 1) was taken as a test case to compare the different ab initio methods [DFT, HF, CCSD, and CCSD(T)], as well as different basis sets that were used. Corannulene has four unique C–C bonds, which are displayed in Figure 2. Geometry optimization calculations, as reported in Table 2, show that the bond lengths calculated by DFT agree very well with the experimental results from the literature.^21^ On the other hand, the other computational methods did not give such accurate results.

**Table 2:** Calculated and Literature Values of C–C Bond Lengths of Corannulene^21^^a^

unique bond	literature(DFT, experimental)	DFT(DZP)	RHF(STO-3G)	CCSD(STO-3G)	CCSD(T)(DZ)
1	1.416, 1415(2)	1.417	1.423	1.457	1.441
2	1.380, 1.379(2)	1.385	1.361	1.394	1.393
3	1.442, 1.446(2)	1.446	1.462	1.491	1.475
4	1.382, 1.383(2)	1.388	1.363	1.403	1.409

a Methods and the basis set employed in the calculations are indicated.

The CNCs of the GA_5_(0, *q*) series, when q is odd, all possess a C_5*v*_ symmetry because they are CS systems. As the cone size increases from corannulene to GA_5_(0, 3) and GA_5_(0, 5), the bond length values of the pentagonal tip slightly increase to 1.422 Å. Beyond this system, there is no difference among the larger cones. The pentagon tip and its surrounding region are very similar among these nanocones, differing only in the case of corannulene. Due to geometrical strain at the tip, the nanocones are characterized by a bowled shape which can be quantified in terms of the apex angle, this point is discussed in the Supporting Information.

The GA_5_(0, *q*) cone series, when q is even, is made of CS^–^ cones, that is, CNCs that possess an open-shell ground-state wave function when neutral and a closed-shell wave function in the anionic form. In the case of these CNCs, the five C–C bonds at the pentagonal tip are no longer equal in length because these molecules undergo Jahn–Teller distortion and do not possess a C_5*v*_ symmetry anymore. The pentagon C–C bond lengths observed for GA_5_(0, 4), as an example, vary from 1.415 to 1.429 Å. After gaining an electron, the CS^–^ nanocones have a closed-shell ground state, the C_5*v*_ symmetry is restored, and the five C–C bonds at the tip are once again equivalent.

The removal of the tip yields a series of GA_5_(1, *q*) cones. As discussed in the Supporting Information Section, the open-tip cones are characterized by a larger bowl shape compared to the GA_5_(0, *q*) nanocones, which could be related to the absence of the strained tip and to the presence of termination hydrogens at the now opened tip that repel each other. The GA_5_(1, *q*) cones have a CS character when *q* is even, and they possess a C_5*v*_ symmetry. On the other hand, they are CS^+^ open-shell systems when *q* is odd and maintain the C_5*v*_ symmetry only in the cation.

**Figure 3:**
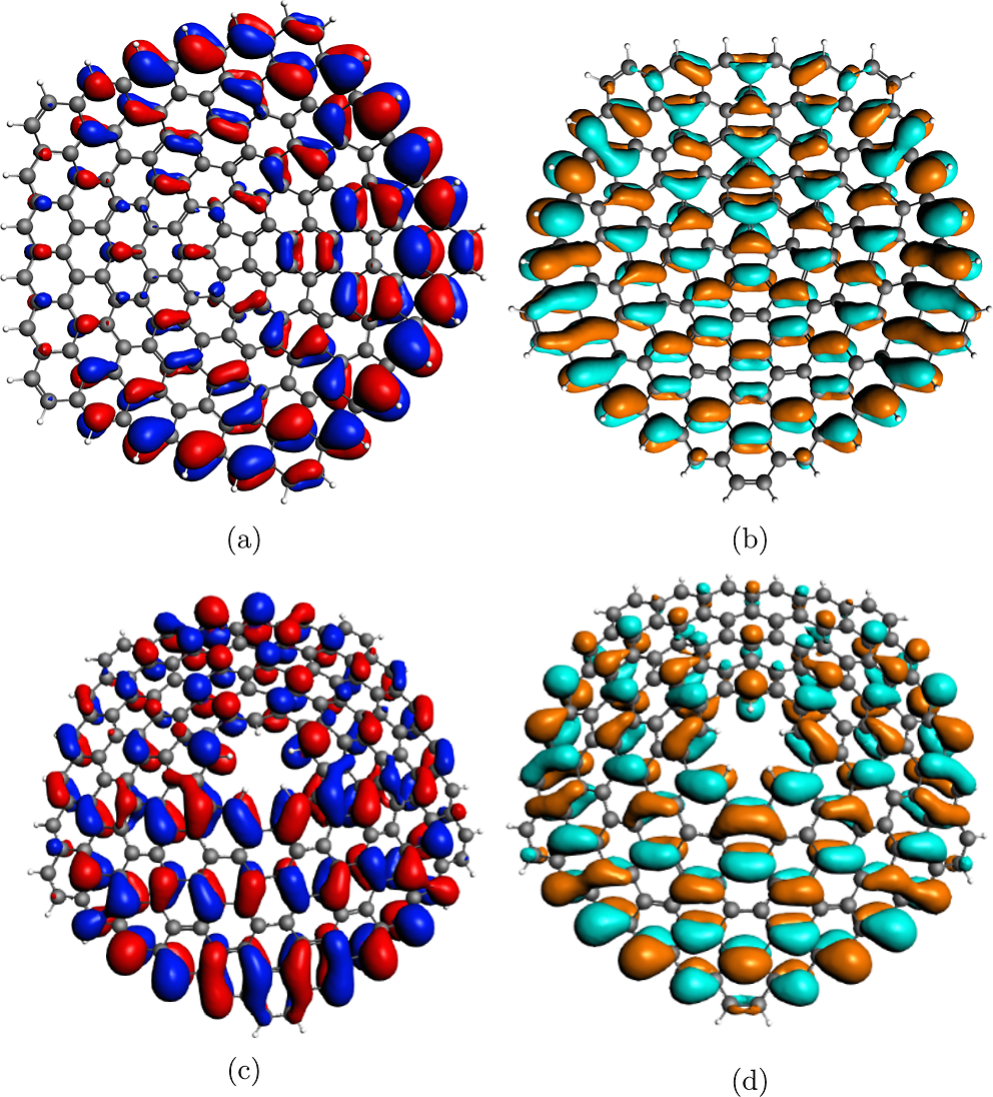
3D plots of: (a) HOMO 75e_2_ of the neutral CS GA_5_(0, 5) and (b)LUMO 76e_1_ of the neutral CS GA_5_(0, 5), (c) HOMO of the neutral OS GA_5_(1, 5), and (d) LUMO of the neutral OS GA_5_(1, 5). Displayed isosurfaces correspond to ±0.01 e^1/2^a_0_^–3/2^ value.

A first insight into the electronic properties of the cones is revealed if we look at the molecular orbitals (MOs), in particular, considering the highest occupied molecular orbital (HOMO) and lowest unoccupied molecular orbital (LUMO). For the GA_5_(0, *q*) closed tip cones, the nature of these MOs changes depending on the wave function character: an odd *q* gives CS character, while an even *q* gives CS^–^ character. As an example, we report in Figure 3 the HOMO and LUMO orbitals of GA_5_(0, 5) and GA_5_(1, 5) cones.

All of the orbitals have a clear π character, as expected. For the CS GA_5_(0, 5) cone (upper panel), the HOMO corresponds to the molecular orbital 75e_2_ accordingly to the C_5_*v* point group symmetry. The localization on one side of the ring is an artifact of the degenerate irreducible representation; in fact, the other component (not reported in the figure) of the doubly degenerate 75e_2_ orbital has an opposite localization which exactly compensates for this effect. On the other hand, it is well apparent that the HOMO displays a very limited localization on the nanocone tip. Furthermore, the LUMO (orbital 76e_1_) is more localized on the pentagonal tip. When q is even, the cone is a CS^–^ open shell, and the symmetry of the system is lost due to the Jahn–Teller effect, the molecule being distorted, as previously described. The HOMO is more localized on the tip compared to the GA_5_(0, 5) HOMO and the LUMO is still localized on the tip as well, suggesting a possible tendency to interact with the electron-donor system in the tip region.

**Figure 4:**
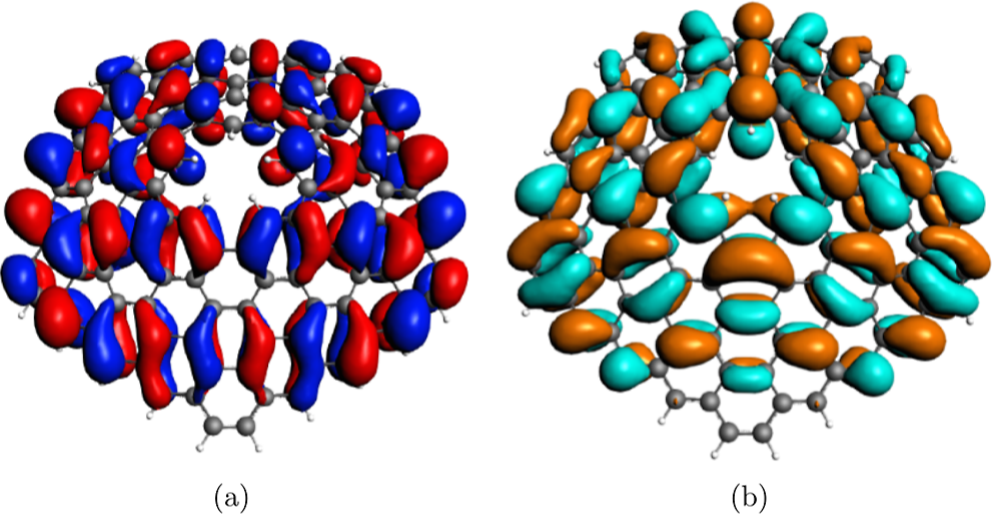
3D plots of: (a) HOMO of the neutral CS GA_5_(1, 4) and (b) LUMO of the neutral CS GA_5_(1, 4). Displayed isosurfaces correspond to ±0.01 e^1/2^a_0_^–3/2^ value.

In the case of the open tip GA_5_(1, *q*) systems, the HOMO and LUMO orbitals appear quite similar irrespective of the CS or CS^+^ character of the wave function. Figure 4 reports the example of HOMO and LUMO of GA_5_(1, 4) (CS) and GA_5_(1, 5) (CS^+^) cones. The LUMO, despite the absence of the pentagonal tip, do not change significantly and still maintain an electron accepting character as observed for the close-tip cones.

### Band Gap (*E*_g_)

The energy difference between HOMO and LUMO is known as the HOMO–LUMO gap or band gap (energy gap, *E*_g_). This energy value is a major factor in determining the electric conductivity of a material, since the conduction electron population *N* of a semiconductor is given by , where *A* is a constant, *k* is Boltzmann’s constant, and *T* is the absolute temperature.^22^ The band gap is highly relevant as a material property because it also influences the efficiency of solar cell and variations in the conductivity can be used for sensing applications;^9,10,23^ moreover, it is one of the simplest characteristics of the electronic structure. Large *E*_g_ values (several eV) are typical of stable closed shell systems (insulators), while a null *E*_g_ implies an open-shell electronic structure (a metallic behavior for solids). Very small *E*_g_ (a few tenths of eV) suggests a semiconductor nature or a situation where the electronic ground state is almost degenerate with low-lying excited states. Besides being a useful descriptor of the electronic structure, *E*_g_ can be also monitored when studying the convergence of finite size systems with increasing size toward the bulk limit.

Before discussing the present *E*_g_ results, it is important to underline that DFT/B3LYP calculations do not accurately predict the frontier orbitals energies of conjugated systems, generally giving less negative HOMO energy (far higher-lying HOMO) and more negative LUMO energy (far lower-lying LUMO) compared to the experimental values. Consequently, the HOMO–LUMO gap results are underestimated. The prediction of this gap can be improved by employing nonempirically tuned range-separated functionals, which significantly increases the accuracy of the computed orbital energies.^24−26^ Because the main purpose of the present study focuses on the relationship among the electronic properties of the nanocones and the GHR ground-state wave function character, we still decided to employ the conventional hybrid B3LYP, which is known to yield accurate geometries of conjugated systems^24^ as well as a reasonably good description of ground-state electronic structure.

Anyway, the *E*_g_ value from the present B3LYP calculations of the smallest curved graphannulene, GA_5_(0, 1) or corannulene, of 4.39 eV agrees very well with literature values of 4.44 eV^23^ and 4.34 eV.^27^ It is worth noting that the GA_5_(0, 1) *E*_g_ has proven very sensitive to doping by B, N, and F atoms.^27,28^ CNCs have semiconductor properties^7^ and as they grow in size, the spacing between energy levels decreases and so does *E*_g_. This dependence of *E*_g_ with size is a general property and has been observed for polyaromatic hydrocarbons (PAHs) and CNTs.^15,29,30^ In Table 3 and 4, the decrease of *E*_g_ with increasing CNC size is quite apparent, notice that these values range from 4.39 eV for the smallest GA_5_(0, *q*) system down to 0.53 eV for the largest system considered. In Tables 3 and 4, empty boxes for *E*_g_ correspond to the open shell electronic structure. The nature of the wave function character, as predicted by GHR, can therefore give a prior indication of the metallic or semiconductor behavior of the nanocone.

**Table 3:** Cohesive Energies and Band Gap of GA_5_(0, *q*) CNCs

	*E*_coh_/atom/eV	*E*_coh_/*N*_C_/eV	HOMO–LUMO Gap/eV
GA_5_(0, 1)	6.20	9.30	4.39
GA_5_(0, 3)	7.00	8.75	2.42
GA_5_(0, 4)	7.20	8.64	
GA_5_(0, 5)	7.35	8.58	1.45
GA_5_(0, 6)	7.46	8.53	
GA_5_(0, 7)	7.54	8.49	0.88
GA_5_(0, 8)	7.61	8.46	
GA_5_(0, 9)	7.67	8.43	0.53

**Table 4:** Cohesive Energies and Band Gap of GA_5_(1, *q*) CNCs

	*E*_coh_/atom/eV	*E*_coh_/*N*_C_/eV	HOMO–LUMO Gap/eV
GA_5_(1, 2)	6.22	9.33	3.43
GA_5_(1, 3)	6.71	8.95	
GA_5_(1, 4)	7.02	8.77	1.96
GA_5_(1, 5)	7.21	8.66	
GA_5_(1, 6)	7.36	8.58	1.22
GA_5_(1, 7)	7.47	8.53	

### Cohesive Energy

The CNC cohesive energy per atom is a measure of relative stability and is defined as the difference between the total energy of the system and of the isolated atoms divided by either the total number of atoms or by the number of carbon atoms. Therefore: *E*_coh_/atom = −(*E*_tot_ – *N*_C_*E*_C_ – *N*_H_*E*_H_)/*N*_total_, or *E*_coh_/*N*_C_ = −(*E*_tot_ – *N*_C_*E*_C_ – *N*_H_*E*_H_)/*N*_C_, where *N*_C_, *N*_H_ and *N*_total_ are the number of C atoms, the number of H atoms, and the total number of atoms, respectively. *E*_tot_ is the CNC total energy, *E*_C_ is the total energy of an isolated C atom, and *E*_H_ is the energy of an isolated H atom. The calculated cohesive energies for CNCs are reported in Tables 3 and 4.

For both the closed and open tip nanocones, the *E*_coh_/atom increases with the increasing CNC size while *E*_coh_/*N*_C_ decreases with the size of the nanocone. Smaller nanocones have a larger proportion of hydrogen atoms than larger nanocones and therefore, a larger proportion of C–H bonds. In the case of GA_5_(0, 1) or corannulene, for example, 1/3 of its atoms are hydrogen while in GA_5_(0, 9) only 1/11 of its atoms are hydrogens. This in turn makes it difficult to compare different nanocones, as they have different compositions. The C–H bonds have a lower bonding energy than the C=C bonds; therefore, molecules with more hydrogens have lower cohesive energies per atom. *E*_coh_/*N*_C_ is logically always larger than *E*_coh_/atom; it decreases with the increasing CNC size because the amount of H atoms contributing to the bonding energy, which are not counted in N_*C*_, decreases. In the literature, the *E*_coh_/atom of CNCs ranges from around 6.2 to 7.4 eV,^13,31^ while the range of *E*_coh_/*N*_C_ is around 8.0–9.9 eV.^13,32^ Although an *E*_coh_/atom value of 7.93 eV presently calculated for GA_5_(0, 1) agrees well with the literature value,^28^ the cohesive energies calculated for the larger nanocones seem larger than expected. Indeed, one has to be careful when analyzing the cohesive energy calculated with DFT because it tends to be overestimated.^33,34^

One interesting trend emerges when comparing the *E*_coh_ of the GA_5_(0, *q*) cones of Table 3 with the GA_5_(1, *q* + 1) ones of Table 4, because these pairs of closed- and open-tip cones have the same C/H ratio in their composition. Indeed, the cohesive energy of these pairs is very similar, with the cohesive energies of the GA_5_(1, *q* + 1) cones being slightly higher than that of the GA_5_(0, *q*) cones. Because the GA_5_(1, *q* + 1) cones have an open tip, they have a smaller strain than the CNCs with the pentagonal tip, leading to slightly more stable structures. *E*_coh_/atom for GA_5_(1, 2) is 0.05 eV higher than that for GA_5_(0, 1). For larger structures, on the other hand, the difference is negligible, this suggests that this geometric strain is localized near the tip and it plays a minor role as the size of the CNC increases.

The possibility to have two different definitions of the cohesive energy (per total atoms or per carbon atom) is not completely satisfactory, so it would be interesting to suggest an alternative definition that does not suffer this ambiguity. A very appealing possibility is to perform a multiple linear regression of the bonding energy (BE_*i*_) with respect to a pair of independent variables: the number of C and H atoms, the fitting coefficients being defined as the cohesive energy of C and H atoms, respectively. The BE_*i*_ is defined as the negative of the formation energy of the *i*-th CNC with respect to the free atoms. In practice, if we index a specific CNC with the *i*-th index, we can fit BE_*i*_ with the following straight plane, which we impose to pass through the origin
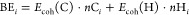
where *E*_coh_(C) and *E*_coh_(H) are the cohesive energy of C and H atoms, respectively, and *n*C_*i*_ and *n*H_*i*_ are the number of C and H atoms contained in the *i*-th CNC.

In order to test the robustness of such a procedure, we have fitted separately the two different CNCs GA_5_(0, *q*) and GA_5_(1, *q*) and obtained the following results
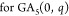

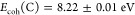

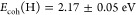

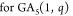

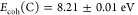

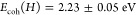


These results are remarkable for many reasons: first of all, very similar results of *E*_coh_ are obtained in practice by fitting separately two independent sets of data, which suggests that this simple linear model is very realistic. Moreover, the *E*_coh_(*C*) = 8.21 eV is consistent with an extrapolation in Tables 3 and 4, because these values lie just between the two definitions of *E*_coh_, taking the C atoms or the total atoms. Finally, the standard deviations are quite small and consistent between the two data sets and the *E*_coh_(H), as expected, is much lower than *E*_coh_(C), by a factor of almost 4.

### Dipole Moment

The presence of a pentagon tip in CNCs induces an excess of charge density, which is consistent with the point effect in electrostatics.^35,36^ This directly relates to the dipole moment of the CNCs because it has been observed that the introduction of curvature into a hexagonal carbon lattice through the inclusion of a pentagon ring produces a considerable dipole moment.^37^ The main origin of this dipole can be traced to flexoelectric polarization, induced by the curvature, of the bonds in the direction normal to the C-skeleton.^37^

Table 5 shows the results obtained for the CNCs considered in this study. The first remark concerns the value of 3.0 D calculated for corannulene, which does not compare well with the experimental value of 2.071 D.^38^ This disparity was already reported in literature when the B3LYP potential was used with smaller basis sets; however, it was shown that the dipole moment converged to 2.044 D when a large basis set was used.^37^ This demonstrates that an accurate description of the dipole moment of CNCs would require basis sets larger than the DZP basis set. Nevertheless, general trends in the presently calculated dipole moments can still be discussed.

**Table 5:** Dipole Moment of CNCs

dipole moment/Debye			
GA_5_(0, 1)	3.0	GA_5_(1, 2)	4.0
GA_5_(0, 3)	9.2	GA_5_(1, 3)	6.2
GA_5_(0, 4)	12.2	GA_5_(1, 4)	8.5
GA_5_(0, 5)	15.3	GA_5_(1, 5)	11.2
GA_5_(0, 6)	18.8	GA_5_(1, 6)	13.9
GA_5_(0, 7)	22.4		
GA_5_(0, 9)	30.5		

**Figure 5:**
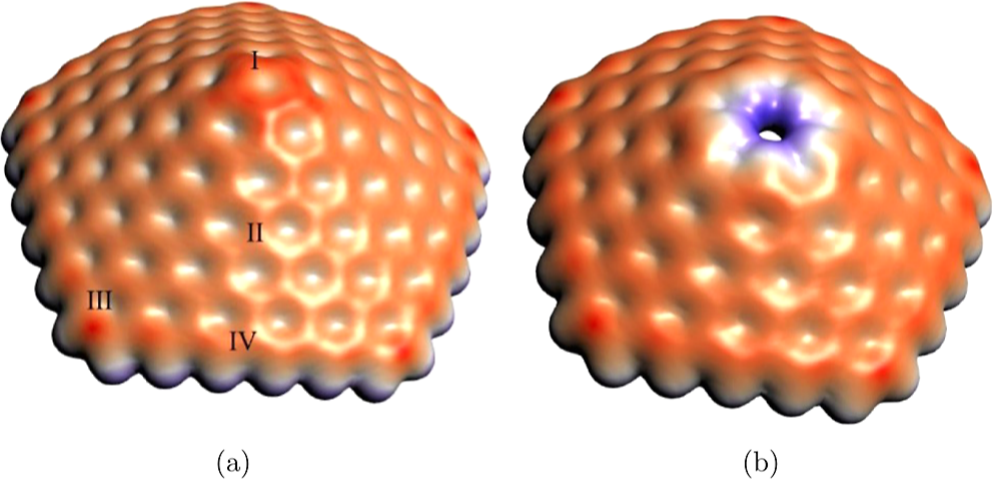
Electrostatic potential of GA_5_(0, 5) (a) and GA_5_(1, 4) (b) CNCs. Isosurface value of ±0.005 e^1/2^a_0_^–3/2^, where red represents negative potentials, and blue represents positive potentials.

A significant dipole moment is calculated for both the closed-tip GA_5_(0, *q*) and open tip GA_5_(1, *q*) series and a strong dependency on the size of the CNCs emerges, which is in agreement with the trends of literature which generally shows a linear increase with the size of the nanocone.^37,39^ The open tip cones have a smaller dipole moment compared with the closed tip ones. This behavior can be ascribed to the reduction of the strain following the removal of the pentagon tip because the dipole moment in CNCs is due to the strain gradient; furthermore, the H atoms at the open tip faced at approximately the opposite direction of the H atoms at the base, leading to a partial cancellation of the dipole moment. The difference in the charge distribution between the closed and open tip nanocones emerges also from the electrostatic potential, as reported in Figure 5 for the GA_5_(0, 5) closed tip and GA_5_(1, 4) open tip cones. An excess of positive charge dominates the potential in the region of the terminating hydrogen atoms, in particular at the open tip (Figure 5b), instead a negative charge dominates in the region around the nanocones, due to the π-electrons of the aromatic system. A great localization of negative charge is present in the region of the pentagon tip, as shown in Figure 5a, due to the flexoelectric effect previously mentioned, which is consistent with the point effect in electrostatics^35,36^ and as extensively reported in the literature.^9,13,32,37^

### Electron Affinity and Ionization Potential

The electron affinity (EA) and ionization potential (IP) are important electronic properties to characterize the CNCs because they allow us to infer the relative likelihood of the system gaining or losing an electron, respectively. Furthermore, it is interesting to analyze the implication of the wave function character of the CNCs, as predicted by the GHR, on these properties, considering that, in general, as a molecule increases in size, it is able to accommodate a charge more easily, leading to higher EAs and lower IPs.

A validation test of the DFT computational approach employed for the calculations of the EAs was performed preliminarily by considering the GA_5_(0, 0) system, which corresponds to the cyclopentadienyl radical, for which the CCSD(T) approach can be also employed. A value of 2.0 eV was obtained at the CCSD(T) level with an extended basis set (triple-zeta plus polarization, complete contractions), which compares well with literature values around 1.8 eV^40,41^ as well as with a DFT value of 1.82 eV, confirming the adequacy of the DFT approach to treat also these electronic properties of graphannulenes.

The DFT calculated EAs and IPs of the CNCs are reported in Table 6 together with the character of the wave function as predicted by the GHR.

**Table 6:** Electron Affinity and Ionization Potential for Different CNCs

electron affinity/eV		ionization potential/eV	
GA_5_(0, 3) CS	2.51	GA_5_(1, 2) CS	6.94
GA_5_(0, 4) CS^–^	3.77	GA_5_(1, 3) CS^+^	5.26
GA_5_(0, 5) CS	3.33	GA_5_(1, 4) CS	5.99
GA_5_(0, 6) CS^–^	3.77	GA_5_(1, 5) CS^+^	5.19
GA_5_(0, 7) CS	3.59	GA_5_(1, 6) CS	5.43

**Figure 6:**
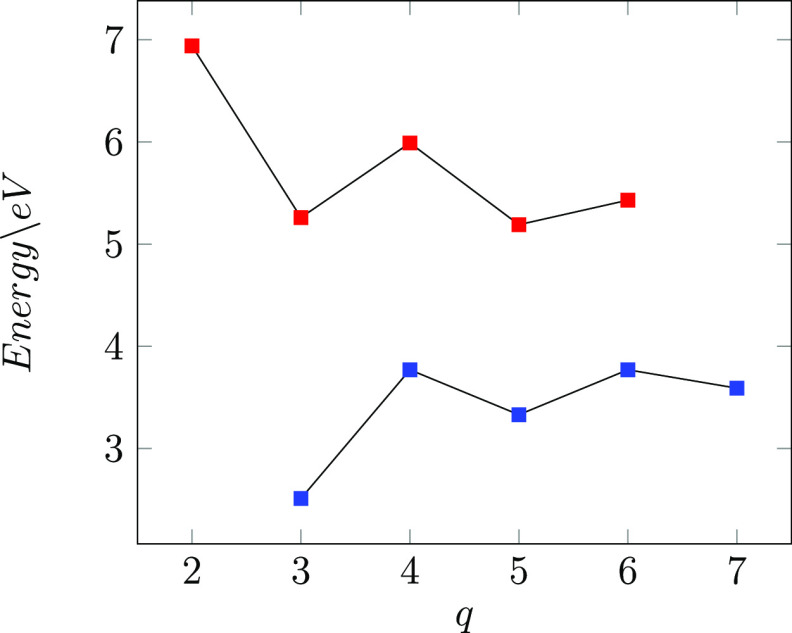
Electron affinity (blue) and ionization potential (red) of GA_5_(0, *q*) and GA_5_(1, *q*) CNCs, respectively.

The EAs were calculated for the GA_5_(0, *q*) cones because to this series belong the CS^–^ cones [GA_5_(0, *q*) with q even], which acquire a CS stable closed-shell wave function when gaining an electron. For this reason, the EAs obtained for the CS^–^ cones are larger than those of the CS ones of the GA_5_(0, *q*) group; furthermore, the expected increase of the EA with the size of the cones is no more respected along the series. An oscillating behavior of the EAs with the increasing size of the GA_5_(0, *q*) cones therefore emerges, as shown in Figure 6. We note that all the GA_5_(0, *q*) cones have a positive electron affinity; therefore, the minimum energy value always corresponds to a negatively charged molecule, instead of the neutral one, as found in fullerenes.^42^

The IPs were calculated for the open tip GA_5_(1, *q*) series because the CS^+^ cones (GA_5_(1, *q*) with q odd) become closed shell systems by losing an electron. The CS^+^ cones have in fact the lowest IP values of the series; therefore, the expected decrease of the IPs along the series with the increasing size of the cones is broken and the IPs trend follows an oscillating behavior (see Figure 6), as found for the EA trend.

**Figure 7:**
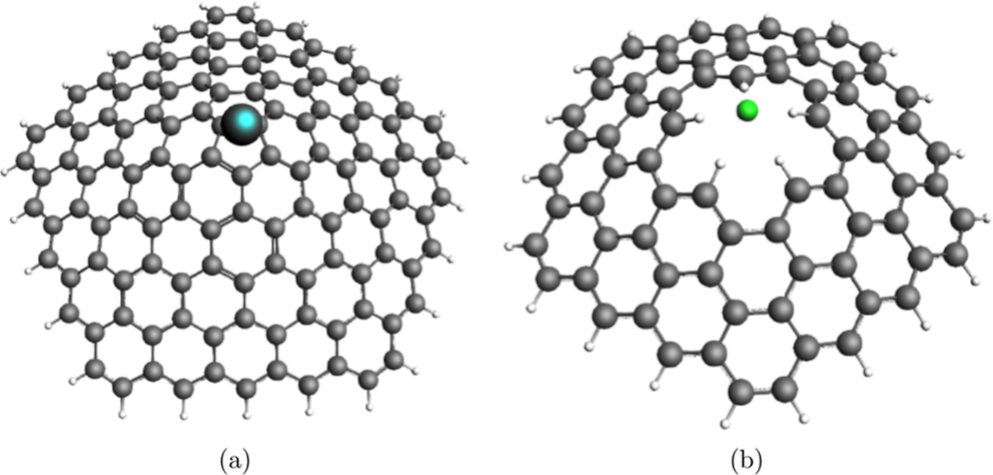
Optimized geometry of CNC-A systems, where (a) A = Li and (b) A = F.

The oscillating behavior of the CNC EAs and IPs indicates that the stability of the ionic forms of the nanocones cannot be related only to their dimensions but it is necessary to take into account the ground-state wave function character as predicted by the GHR for any particular CNC. Oscillation of these properties with the increase in the system size has been also observed in CNTs and in graphene nanodots.^29,43−45^

### Adsorption of Li and F

The implication of the ground-state wave function character of the CNCs, as predicted by the GHR, can be also investigated by considering the adsorption of lithium on GA_5_(0, *q*) cones and fluorine on GA_5_(1, *q*) ones. It has been demonstrated that the adsorption of different species preferentially occurs on the apex of CNCs.^9,10,13^ Indeed the molecular electrostatic potential of the external surface of GA_5_(0, *q*) cones (Figure 5 a) indicates that a negative charge accumulates around the pentagon on the apex. Furthermore, the LUMO of these cones has an electron-accepting character, as previously discussed. This site represents therefore an active site for the interaction with an electron donor atom, such as a Li atom. Instead, the C–H terminal bonds at the tip of the GA_5_(1, *q*) open cones are slightly polarized, and the consequent increased positive partial charge on the hydrogen atoms (as shown in Figure 5b) enables the conditions for the reaction with the electronegative fluorine atom.

The resulting distance between Li and one of the C atoms of the pentagonal tip is 2.3 Å, while in the case of F the distance to the hydrogens of the open tip is 1.8 Å. The calculated atomic partial (Hirshfeld atomic charge and Voronoi deformation density) charge of Li in the GA_5_(0, 4)-Li cone was calculated to be 0.514 and 0.532 *e*, while fluorine in the GA_5_(1, 3)-F cone presented atomic partial charges of −0.383 and −0.464*e*, respectively. The inspection of the partial atomic charges shows that a considerable charge transfer occurs between the CNCs and A and, therefore, the adsorbate systems can be thought of as being represented by CNC^–^-Li^+^ or CNC^+^-F^–^.

Table 7 collects the adsorption energies (*E*_ads_) of GA_5_(0, *q*)-Li and GA_5_(1, *q*)-F cones; the GS wave function character of the cones without the adsorbed atom, as predicted by the GHR, is also reported. The values of the adsorption energy indicate an effective interaction of both Li and F atoms with the respective series of CNCs; however, the *E*_ads_ values are significantly different for the two series, being around or lower than 50 kcal/mol for the CNC–Li systems and with almost doubled values for the CNC–F cones. This indicates a stronger interaction of the F atom with the C–H dangling bonds of the open tip cones compared to the Li interaction with the pentagonal ring of the GA_5_(0, *q*) cones, confirming the efficiency of the extreme electronegative F atom to react with the positive hydrogens of the open tip.

**Table 7:** Adsorption Energies of Li and F for Different CNCs with Their Respective HOMO–LUMO Gaps

*E*_ads_ of Li[kcal/*mol*]		HOMO–LUMO gap/eV	*E*_ads_ of F[kcal/*mol*]		HOMO–LUMO gap/eV
GA_5_(0, 3) CS	29.63		GA_5_(1, 3) CS^+^	106.13	1.89
GA_5_(0, 4) CS^–^	53.28	1.80	GA_5_(1, 4) CS	84.55	
GA_5_(0, 5) CS	37.82		GA_5_(1, 5) CS^+^	101.29	1.23
GA_5_(0, 6) CS^–^	45.20	1.10	GA_5_(1, 6) CS	93.64	
GA_5_(0, 7) CS	41.78		GA_5_(1, 7) CS^+^	87.40	0.70

The character of the ground-state wave function of the CNCs provides a trend in the adsorption energies of each series: larger Li adsorption energies are found for the CS^–^ GA_5_(0, *q*) cones compared to the CS counterparts, whereas for the F adsorption, the CS^+^ GA_5_(1, *q*) cones have the larger adsorption energies. This trend agrees with that observed of the EAs and IPs, where the CS^–^ GA_5_(0, *q*) cones have a larger tendency to gain an electron than the CS cones, while CS^+^ GA_5_(0, *q*) cones have a larger tendency to lose an electron than the CS counterparts. For this reason, the *E*_ads_ does not increase regularly along each series with the system size rather an oscillating behavior emerges, as already found for the EA, IP, and *E*_g_ properties.

The adsorption of the Li and F atoms alters the electronic properties of the CNCs, in particular it influences the HOMO–LUMO gap, which can be predicted on the basis of the wave function character. In particular, the GA_5_(0, *q*) cones with a CS^–^ character, which have no HOMO–LUMO gap (see Table 3), change to neutral CS GA_5_(0, *q*)-Li systems acquiring semiconductor properties while the GA_5_(0, *q*) CS cones became neutral OS GA_5_(0, *q*)-Li cones switching to a metallic behavior. For the GA_5_(1, *q*) series, the adsorption of F changes the cone with CS^+^ wavefunction character to neutral CS cones, which acquire a band gap, while the GA_5_(1, *q*) CS cones change to neutral GA_5_(1, *q*)-F OS systems without energy gap. The HOMO–LUMO gap decreases as the size of the CNC–Li and CNC–F cones increases, as found for the CNC cones of Table 1 and in general for polyaromatic hydrocarbons (PAHs) and CNTs.^15,29,30^

## Conclusions

In this work, we carried out a systematic DFT study of the electronic properties of closed-apex and open-apex graphannulene systems of different sizes. The optimized geometries indicate a bowl shape for both the close tip GA_5_(0, *q*) and open tip GA_5_(1, *q*) cone series, and the generalized Hückel rule (GHR) is applied to predict the ground-state wave function character of the system. The main focus is to relate the calculated electronic properties of the nanocones to the ground-state wave function character, as predicted by the GHR. For the closed tip GA_5_(0, *q*) cones, we found that the nature of the HOMO and LUMO orbital changes depending on the CS or CS^–^ wave function character, while the removal of the tip in the GA_5_(1, *q*) cones make these orbitals similar irrespective the CS or CS^+^ character of the wave function. The calculated electron affinity, ionization potential, and the Li or F adsorption energy present a close connection to the ground-state wave function character; in particular, a peculiar oscillatory energy trend emerges with respect to the increase of the system sizes for both the closed and open tip cones. The EA and IP oscillating behavior points to a dependency of the ionic forms’ stability not only on the dimension of the cones but also on the ground-state wave function characters. The HOMO–LUMO gap (*E*_g_), the dipole moment, and the cohesive energies (*E*_coh_) are instead properties which depend on the size of the nanocone as well as on the presence/absence of the pentagonal tip. Concerning the *E*_g_, the presence of a band gap can be predicted on the basis of the ground-state wave function character of the cones: the CS character indicates a semiconductor behavior while the open shell character (CS^+^ or CS^–^) indicates a metallic behavior of the nanocones. A trend of the calculated *E*_coh_ with the cone size is found in both series; however, the *E*_coh_ dependency on the composition of the cone makes these analyses not straightforward because it is possible to define the cohesive energy with respect to all atoms or only with respect to the C atoms. Of course the limit for large systems would be the same, but at finite size, as the present series, this ambiguity still persists. In order to overcome this ambiguity intrinsic in the definition of cohesive energies, we tried a multiple linear regression of the total bonding energy with respect to two independent variables: the number of C and H atoms. This procedure has furnished the bonding energies of 8.22 ± 0.01 eV and 2.17 ± 0.05 eV, respectively, for C and H in the GA_5_(0, *q*) series, with a remarkable small standard deviation, confirming the validity of the regression procedure. Almost identical values are found for the GA_5_(1, *q*) series. Moreover, the value obtained for the C atom is in line with those reported in the literature, confirming that the level of theory adopted in the DFT calculations is adequate for an accurate description of the electronic structure of these systems. In summary, carbon nanocones have proven interesting systems with tunable properties by playing on their shape and size, with the GHR representing a useful tool to design them or interpret their behaviors.

## Supporting Information Available

Geometry of nanocones and apex angles (PDF)
