# Supplementary material for: Electronic Structure of Pentagonal Carbon Nanocones: An ab Initio Study
Source: J Phys Chem A. 2023 Nov 8;127(46):9723–32. doi: 10.1021/acs.jpca.3c05062 (PMC10683015; doi:10.1021/acs.jpca.3c05062)

Supplementary Information The Electronic Structure of Pentagonal Carbon Nanocones: an ab-initio Study

Samuel Henrique Mattoso,† Véronique Brumas,‡ Stefano Evangelisti,‡ Giovanna Fronzoni,† Thierry Leininger,‡ and Mauro Stener*,†

† *Dipartimento di Scienze Chimiche e Farmaceutiche, University of Trieste, Via Giorgieri 1, 34127 Trieste - Italy*

‡ *Laboratoire de Chimie et Physique Quantiques - FeRMI, Université de Toulouse 3 (Paul Sabatier) et CNRS, 118, Route de Narbonne, F-31062 Toulouse Cedex - France*

E-mail: stefano.evangelisti@univ-tlse3.fr,stener@units.it

Supporting Information

The geometry optimizations showed that corannulene has a bowl shape, as observed in the literature.[1,2] For the larger cones, a similar behavior was observed. The apex angle θ of the $GA_5(0, q)$ cones was calculated using the distance between several carbon atoms that would be contained in the mathematical cone surface. As seen in Figure S1, the sides of the ABC triangle are measured, using the CNCs optimized geometries. The angle α is then determined using the law of cosines and finally θ is calculated, where it is given by $\theta= 180^o - 2\alpha$.

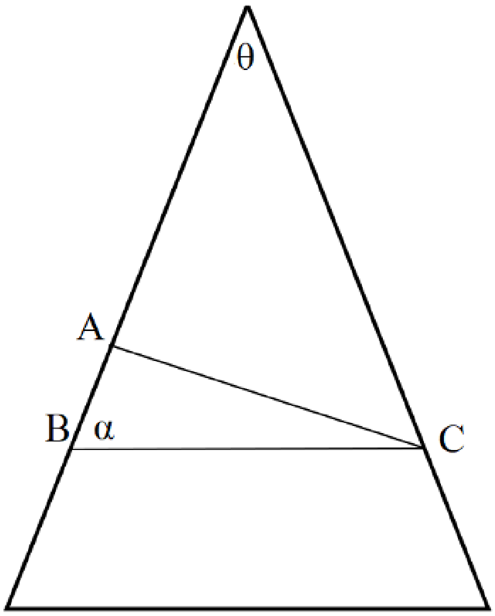

Figure 1

Figure S1: Level surface of a cone for the determination of α and θ angles

Geometrically equivalent triangles with the same proportions can be taken at any height of an ideal cone, each of them having the same value of α and therefore of θ. In the case of the CNCs, however, this was not observed, meaning that different values of θ were found depending on the ABC triangle that was used, showing that the CNCs have indeed a bowl shape. This procedure of calculating different values of θ depending on the triangle taken at a certain height of the CNC provides a way to analyze how much the optimized geometry

departs from an ideal cone shape into a curved or bowl shape. This pattern, of the tip of CNCs having slightly different apex angles compared to the main part, has been suggested in an experimental study by Jaszczak et al., where the geometry of naturally occurring CNCs was analyzed through electron microscopy techniques.[3]

Carbon nanocones with one pentagon at the tip should have ideally an apex angle of θ ideal $= 112.9^o$. In the case of the $GA_5(0, q)$ nanocone, when the xyz coordinates of carbon atoms adjacent to the pentagon tip were used to calculated θ, a value of 122.5^o was obtained. When, however, atoms present on the outermost ring was used, a value of 110.5^o was obtained. This shows that the carbon atoms do not project perfectly onto an ideal conical surface, but that at the tip there is a wider opening as compared to the outer rings, probably due to the strain imposed by the pentagon tip. In Table S1 it is possible to observe the value of the apex angle , which was calculated for three CNCs using the law of cosines. The angles were calculated in three different regions of the CNCs; at the tip, at an intermediate region given by the third layer of the correspondent triangulene fragment and at the base. It is possible to see, for all three CNCs, that $\theta_{tip} > \theta_{ideal}$ while $\theta_{intermediate} \approx \theta_{ideal}$.

Table S1: Values of the apex angle θ of different-size CNCs calculated in three different regions.

	$GA_5(0, 3)$	$GA_5(0, 5)$	$GA_5(0, 7)$
θ_{tip}	121.5°	124.7°	123.3°
$\theta_{intermediate}$	110.6°	111.8°	109.4°
θ_{base}	110.6°	103.3°	109.5°

This shows that indeed the tip has a wider opening, while after a few layers, the geometry approaches the ideal shape. For the $GA_5(0, 3)$ cone, $\theta_{base} = \theta_{intermediate}$ since they were both calculated with the same points. As the CNC grows to $GA_5(0, 5)$, we have that $\theta_{base} < \theta_{ideal}$, demonstrating that its geometry resembles in fact a bowl shape. In the case of $GA_5(0, 9)$,θ_{base} $= \theta_{ideal}$, showing that an increase in size led to a stabilization of the conical shape after the tip.

With the $GA_5(1,4)$ nanocone, in which the pentagon ring has been removed, a different behavior was observed. This nanocone presented a 110.5^o apex angle calculated with the uppermost carbon atoms and a 113.8 ^{o} apex angle calculated with the outermost carbon atoms. This is contrast with the $GA_5(0,q)$ nanocones, where smaller angles were observed at the outer carbon rings. This shows that the removal of the pentagon does affect the geometry of the nanocone, giving a wider base. This could be related to the absence of the strained tip and to presence of termination hydrogens at the now opened tip that repel each other, leading to a cone with a wider base.

References

(1) Karadakov, P. B. Magnetic Shielding Study of Bonding and Aromaticity in Corannulene and Coronene. *Chemistry* **2021**, *3*, 861–872.

(2) Obayes, H. R.; Alwan, G. H.; Al-Amiery, A. A.; Kadhum, A. A. H.; Mohamad, A. B. Thermodynamic and theoretical study of the preparation of new buckyballs from corannulene, coronene, and circulene. *Journal of Nanomaterials* **2013**, *2013*, 1–8.

(3) Jaszczak, J. A.; Robinson, G. W.; Dimovski, S.; Gogotsi, Y. Naturally occurring graphite cones. *Carbon* **2003**, *41*, 2085–2092.

TOC Graphic

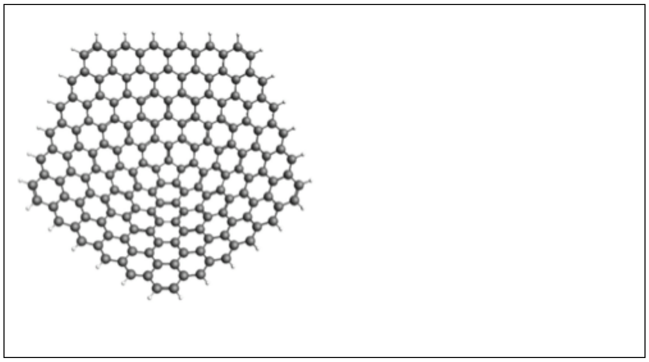

Supplement: Supplementary file 1 — jp3c05062_si_001.pdf [file jp3c05062_si_001.pdf]
